# Comparative analysis, distribution, and characterization of microsatellites in Orf virus genome

**DOI:** 10.1038/s41598-020-70634-6

**Published:** 2020-08-17

**Authors:** Basanta Pravas Sahu, Prativa Majee, Ravi Raj Singh, Anjan Sahoo, Debasis Nayak

**Affiliations:** 1 Discipline of Biosciences and Biomedical Engineering, Indian Institute of Technology Indore, Indore, MP 453 552 India; 2 College of Veterinary Science and Animal Husbandry, Bhubaneswar, 751003 India

**Keywords:** Data mining, Pox virus

## Abstract

Genome-wide in-silico identification of microsatellites or simple sequence repeats (SSRs) in the Orf virus (ORFV), the causative agent of contagious ecthyma has been carried out to investigate the type, distribution and its potential role in the genome evolution. We have investigated eleven ORFV strains, which resulted in the presence of 1,036–1,181 microsatellites per strain. The further screening revealed the presence of 83–107 compound SSRs (cSSRs) per genome. Our analysis indicates the dinucleotide (76.9%) repeats to be the most abundant, followed by trinucleotide (17.7%), mononucleotide (4.9%), tetranucleotide (0.4%) and hexanucleotide (0.2%) repeats. The Relative Abundance (RA) and Relative Density (RD) of these SSRs varied between 7.6–8.4 and 53.0–59.5 bp/kb, respectively. While in the case of cSSRs, the RA and RD ranged from 0.6–0.8 and 12.1–17.0 bp/kb, respectively. Regression analysis of all parameters like the incident of SSRs, RA, and RD significantly correlated with the GC content. But in a case of genome size, except incident SSRs, all other parameters were non-significantly correlated. Nearly all cSSRs were composed of two microsatellites, which showed no biasedness to a particular motif. Motif duplication pattern, such as, (C)-x-(C), (TG)-x-(TG), (AT)-x-(AT), (TC)- x-(TC) and self-complementary motifs, such as (GC)-x-(CG), (TC)-x-(AG), (GT)-x-(CA) and (TC)-x-(AG) were observed in the cSSRs. Finally, in-silico polymorphism was assessed, followed by in-vitro validation using PCR analysis and sequencing. The thirteen polymorphic SSR markers developed in this study were further characterized by mapping with the sequence present in the database. The results of the present study indicate that these SSRs could be a useful tool for identification, analysis of genetic diversity, and understanding the evolutionary status of the virus.

## Introduction

Contagious ecthyma or Orf is a zoonotic viral disease of sheep, goats, and other small ruminants characterized by proliferative skin lesions in and around the oral cavity in the form of erythematous macule, papule, vesicle, pustule, and scabs. The causative agent is the Orf virus (ORFV), a member of the genus Parapoxvirus of the Poxviridae family. The virus is highly contagious, quite stable in the environment, and remains in the infectious form in wools or animal excreta for months to years^1^. The disease is manifested by proliferative lesions on the mouth and muzzle that usually get resolved in 1–2 months^2^. These facial and oral lesions in lambs may interfere with suckling, while lesions on the udder may interfere in feeding neonates. Similarly, foot lesions often cause transient lameness in infected animals, and together all these results in poor health and loss of body weight. Lesions progress through all clinical stages but are generally non-proliferative and usually resolve within 2–3 weeks. ORFV specific antibodies do not seem to confer protective immunity, although the IgG2 isotype is believed to provide some defense against ORFV infection^3^. As IgG2 is not secreted in the colostrum of ruminants, lamb and kids don’t get required protection^4^. Although Orf is normally non-fatal in adults, often comes with high morbidity (up to 100%). While in neonates, Orf can be life-threating as it interferes with suckling of milk from the infected udder or predisposing the animals to the secondary bacterial or fungal infections^5^. For these reasons, the mortality rate may reach up to 15%^6^. There is increasing evidence of ORFV to cross‐infect other species of animals such as camels, gazelles, reindeers, musk ox, and Japanese serows^3^.

The virus can infect humans, particularly those who are closely associated with animal handling. Zoonosis occurs most frequently during lambing, shearing, docking, drenching, or slaughtering of affected animals^1,3^. Orf infections in humans appear in hand^7^ but occasionally seen in the face^8^, nose^9^, axilla^10^, scalp^11^, genitals^12,13^, urethral^8^, and pericanthal eyelid skin and the wound heals spontaneously. However, in immunosuppressive individuals, large-sized poorly healing lesions could remain for an extended period up to a couple of months^14^. This possesses a significant health risk to animal-handlers and veterinarians who often get infected by direct contact and develop painful pustular lesions in the skins. Complications of Orf with secondary bacterial infections are potentially life-threatening and need urgent medical attention.

The ORFV is a classic epitheliotropic virus, having a double-stranded DNA genome with a higher (64%) GC content^15^. The genome consists of central conserved and terminal variable domains with size varying from 134 to 139 kbp having ~ 130 putative genes, 88 of which are conserved to Chordopoxviruses^16,17^. Having such a devastating character, this virus has got less attention in terms of genomic information, which is evident from the availability of only eleven complete genome sequences worldwide. Several conserved genomic regions such as envelope protein B2L (ORFV011), F1L (ORFV059), and A32L (ORFV108) were used for ORFV identification and phylogenetic tree construction^18^. Still, there is a lack of clarity regarding the real diversity of ORFV due to the absence of a reliable system for virus identification, which consists of hypermutable regions such as microsatellites rather than conventional conserved genes.

Simple sequence repeats (SSRs), also known as microsatellites, refer to mono-, di-, tri-, tetra-, penta- and hexanucleotide sequence units that are repeated in tandem in a genome^19^. Those short motifs of DNA are distributed ubiquitously in the genome of eukaryotes^20^, and prokaryotes^21^, and is regarded as the most variable type of DNA sequence within the viral genome^22,23^. The microsatellites may be classified as either simple or compound, depending on the constituent of nucleotide sequences. The interruptions present in the microsatellite will give rise to interrupted pure, compound, interrupted compound, complex and interrupted complex types. Two or more microsatellites resides directly adjacent to each other to form compound microsatellites by interruption of repeats^24^. Due to its unique characteristics, these SSRs play a major role in meiotic recombination^25–27^, the evolution of species^28^, genome mapping^29^, differentiation of viral strains^30^, studying population genetics^31^, and secondary structure formation^32^. Many studies have highlighted the presence of microsatellite repeats in viruses, such as menovirus^33^, vesicular stomatitis virus^34^, hepatitis C virus^35^, and human respiratory syncytial virus (RSV)^36^. Here, we report for the first time a comparative analysis of microsatellites with respect to the abundance, distribution, composition, and polymorphism of SSRs within ORFV through in-silico approach, followed by the development and characterization of thirteen microsatellites markers. Using these tools, we further tested its usefulness by screening the viral genome from an ORFV outbreak and constructing a concatenated phylogenetic tree, which elucidated that the investigated virus closely related to the Chinese isolate. These markers could be used as a tool for making multiplex PCR assays for virus identification, strain demarcation, and evolutionary analysis.

## Materials and methods

### Genome sequences

The publicly available eleven complete genome sequences of ORFV isolates obtained from the NCBI database (www.ncbi.nlm.nih.gov) were used for genome-wide in-silico microsatellites analysis. To compare genomic sequences of different lengths, we calculated the Relative Density (RD) and Relative Abundance (RA) values. RD is defined as the total length (bp) contributed by each microsatellite per kilobase (kb) of sequence analyzed whereas; RA is the number of microsatellites present per kb of the genome (kb). Among all the strains, we have chosen OV-SA00 (Acc. number: AY386264) as the reference to evaluate the polymorphism of microsatellites through in-silico approach as well as the development of SSRs for Indian origin ORFV (Table 1).

**Table 1:** Overview of microsatellites in ORFV complete genome sequences.

Sr. no.	Acc. no.	Names of the strains	Year of strain isolation	Size (bp)	Country	Host	GC content (%)	Total no of SSRs	RA	RD	Total no of cSSRs	cRA	cRD	% of cSSR
S1	AY386264	OV-SA00	2004	139,962	USA	Goat	63.44	1,181	8.43	59.5	107	0.76	16.98	9.06
S2	AY386263	OV-IA82	2004	137,241	USA	Lamb	64.33	1,089	7.93	55.66	98	0.67	14.51	8.99
S3	DQ184476	NZ2	2006	137,820	New Zealand	Sheep	64.34	1,082	7.85	55.42	95	0.68	14.11	8.78
S4	HM133903	D1701	2011	134,038	Germany	Sheep	63.69	1,038	7.74	54.34	83	0.61	12.13	7.99
S5	KF234407	NA11	2015	137,080	China	Sheep	63.63	1,049	7.65	53.54	87	0.63	12.78	8.29
S6	KP010353	YX	2015	138,231	China	Goat	63.75	1,099	7.95	55.4	90	0.65	12.89	8.18
S7	KP010354	GO	2018	139,866	China	Goat	63.6	1,114	7.96	55.61	97	0.69	13.81	8.7
S8	KP010355	NP	2015	132,111	China	Goat	63.76	1,054	7.97	56.02	86	0.65	12.8	8.15
S9	KP010356	SJ1	2015	139,112	China	Goat	63.63	1,126	8.09	57.01	99	0.71	13.74	8.79
S10	KY053526	OV-HN3/12	2012	136,643	China	Sheep	63.67	1,036	7.58	53.04	84	0.61	12.31	8.18
S11	MG712417	SY17	2016	140,413	China	Sheep	63.81	1,087	7.74	54.28	92	0.65	12.97	8.46

### Microsatellites identification, investigation, and statistical analysis

For identification of perfect mono, di, tri, tetra, penta, hexa as well as compound microsatellites, IMEx software^37^ was utilized. Microsatellites from genomes were extracted using the ‘Advance-Mode’ of IMEx using the parameters previously used for RNA viruses^38,39^ and DNA viruses^40^. The parameters used were as follows: type of repeat: perfect; repeat size: all; minimum repeat number: 6, 3, 3, 3, 3, 3 for mono, di, tri, tetra, penta and hexanucleotide repeats, respectively. The maximum distance allowed between any two SSRs (dMAX) is 10 nucleotides. Other parameters were used as default. Compound microsatellites (cSSRs) were not standardized in order to determine real composition.

### Multiple sequence alignment and identification of polymorphic SSRs

The microsatellites of OV-SA00 were considered for the identification of polymorphic microsatellites as well as consensus motifs. Sequences were first transferred to BioEdit version 7.2.5 software^41^ and aligned by CLUSTAL W^42^ module and checked manually for the presence of polymorphism. The Circos plot was generated using the Circos software to map the genome size, CDS, SSRs distribution, cSSRs distribution, and GC content in ORFV (OV-SA00) genome.

### Disease outbreak and sample data collection

**Figure 1:**
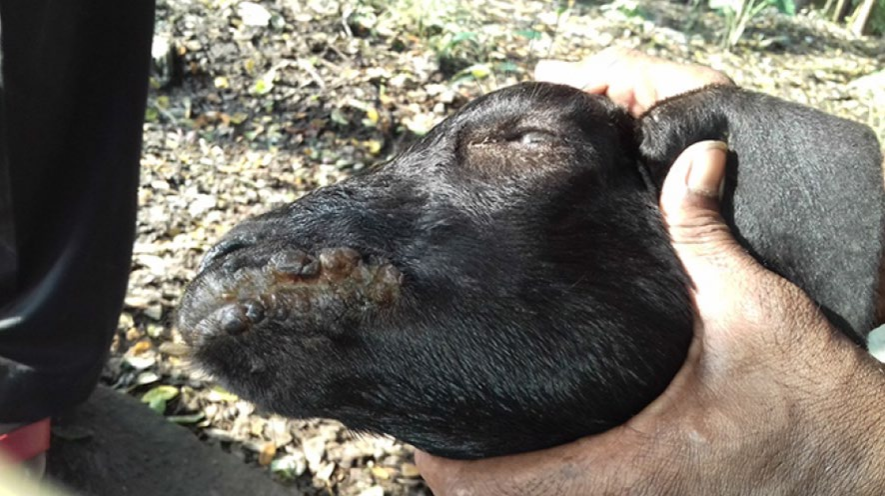
ORFV infection in goat. Representative figure depicting clinical cases of ORFV infection in Black Bengal goat having proliferative lesions around the lip recorded in the study area.

The study did not involve experiments on live vertebrates. Rather, samples were collected from the diseased goats (showing the symptoms of Orf) those reported for veterinary care where scab samples were collected by veterinary professionals as a routine practice. In October and November 2017, an outbreak of ORFV was noticed in Black Bengal goats in the Eastern-Indian state of Odisha with the geographical location (20.4625° N, 85.8830° E). Tissue samples in the form of scabs from four suspected goats were collected at both infective and recovery/convalescent phase and simultaneously treated for wounds with 2% boro glycerine and parenteral application of Enrofloxacin @ 5 mg/kg IM (Fig. 1).

About 5 g of tissue samples were collected from each animal and subsequently dissolved in phosphate-buffered saline (PBS, pH 7.2) added with antibiotics and antifungal supplements in a labeled sterile tube. The homogenized samples were then treated with tissue lysis buffer containing proteinase K, and the mixture was incubated at 56 °C overnight. Finally, the mixture was passed through a column, and DNA was purified from the column by using the standard phenol–chloroform method as described by Sambrook et al.^43^ and stored at − 20 °C until further use. The suspected samples collected during this outbreak produced the expected PCR-amplified fragment size of 140 bp using ORFV specific primers orf1 and orf2^44^ having nucleotide sequences Orf1: 5′-CGCAGACGTGGCTGAGTACGT-3′ and Orf2: 5′-TGAGCTGGTTGGCGCTGTCCT-3′, which confirmed the presence of the virus.

### Development of polymorphic SSRs

The polymorphic microsatellites identified through in-silico approach were further validated through in-vitro approach using ORFV positive clinical sample. Motifs located within defined flanking regions were PCR amplified using specially designed SSR-PCR primer pairs by Primer3Plus web tool (https://www.bioinformatics.nl/cgi-bin/primer3plus/primer3plus.cgi/). The primer length was kept between 18 and 22 bp with product size in the range of 130–200 bp. For proper annealing to the template DNA, the annealing temperature was adjusted between 54 and 61 °C. The thermal cycling conditions for all genes were as follows: initial denaturation step at 95 °C for 5 min, with 35 cycles of denaturation at 95 °C for 50 s, with varying annealing temperature for each set of primers (55–61 °C) and extension step at 72 °C for 90 s with a final extension at 72 °C for 7 min. PCR amplification was performed in a Thermal Cycler system 2,720 (Applied Biosystems, USA) (Table 2).

**Table 2:** Characteristics of the 13 microsatellite markers developed for the ORFV.

Primer name	Sequence	Expected size (bp)	Target repeat	Functional region of the genome	ORF	Position in genome	Temp (°C)	No. of variants
ORFV-SSR-1	F-CACCACCATTAACACCACCA R-AAAGGGTTCGCAAGTACACC	166	(CA)_3_	Hypothetical protein	ORF005	4,974–4,979	55	2
ORFV-SSR-2	F-GACCGTGGCGAGATCCAC R-CACCCTTATTGCCATTCAGC	159	(GGC)_3_	Ankyrin repeat protein	ORF008	7,290–7,298	55	2
ORFV-SSR-3	F-ATCTTTATGGGCGCTGAATG R-CCCAGTGTAGAGGCCAATTC	151	(A)_7_	Intergenic region		7,406–7,412	56	3
ORFV-SSR-4	F-ATGAGCACAATGCAGACCAG R-GAGCAGACACTGCCTACGAC	130	(CG)_3_	Hypothetical protein	ORF015	13,445–13,450	58	2
ORFV-SSR-5	F-TCAAAGTCCTCGTCCGAGTT R-CACATTCACCGAGGAGCAG	168	(TAC)_3_	DNA-binding phosphoprotein	ORF032	34,352–34,360	56	2
ORFV-SSR-6	F-ATGACCTAGAGCCCGTGGAC R-GAGCAGGTCATTCGTGGAG	172	(GAG)_3_	Virion core protein	ORF088	93,996–94,004	55	2
ORFV-SSR-7	F-GCCGCCACTACTTCAGAAAC R-CTAGAGCCAGCGCAGGTACA	200	(T)_6_	Intergenic region		117,434–117,439	60	2
ORFV-SSR-8	F-TTTACGTGAAGGCGTTCCT R-TGAGGCACTTCCTGGACATC	159	(A)_6_	GM-CSF/IL-2 inhibition factor-like protein	ORF117	118,261–118,266	58	2
ORFV-SSR-9	F-TTCCTAGGTGCGTTCAGAGG R-GAGCTGTCGGGGATCTCG	155	(CAC)_3_	Ankyrin repeat protein	ORF121	121,158–121,166	54	2
ORFV-SSR-10	F-TCACTACGAGACCCCTGACC R-AGTGCTTCATTGGGAAGTCG	164	(C)_6_	Ankyrin repeat protein	ORF121	121,625–121,630	61	2
ORFV-SSR-11	F-CACAGATGCGTATTGTGTTGAG R-TTCAGTTGGTCTTTCATCTGGA	156	(AGT)_3_	IL-10-like protein	ORF127	128,736–128,744	57	2
ORFV-SSR-12	F-AGTTATCGGTCGGATTCTCG R-GCGCAATACGAGAGTGAACA	150	(AGTTAC)_3_	Intergenic region	–	129,259–129,276	55	3
ORFV-SSR-13	F-GTTCTCCCGCTGGATAAATG R-CGAGGAAGACGTCGTACAGC	160	(CGC)_3_	Putative serine/threonine protein kinase	ORF130	134,033–134,041	55	2

The amplified products were resolved by electrophoresis in a 3% agarose gel. The PCR amplified products, stained with ethidium bromide, were visualized and photographed using a Gel Doc™ XR + System with Image Lab™ Software (Bio-Rad®). Subsequently, the amplified products were purified using QIAquick® purification kit (QIAGEN, USA) and the purified fragments were sent for sequencing using 3100 ABI sequencer (Applied Biosystems, USA) as described by Sanger et al.^45^. All sequences obtained were analyzed and verified twice in each direction.

### Sequencing data analysis and phylogenetic tree construction

The sequencing results of the developed SSR markers were aligned by using discontiguous-MegaBLAST to identify specific regions among the reads (microsatellites) within the ORFV genome^46^. Next, the sequencing results were subjected to the BLASTx analysis, which compares translational products of the nucleotide query sequence to protein databases (https://www.ncbi. nlm.nih.gov). A concatenated phylogenetic tree was constructed using the bootstrap consensus tree building method of neighbor-joining with bootstrap value 500 through MEGA 5 to elucidate the genetic relationship of the outbreak sample with the global strains of ORFV.

## Results

### Distribution of SSRs and cSSRs in ORFV genome

Our study revealed a large number of SSRs scattered throughout the ORFV genomes varying from 1,036 to 1,181 in number with an average of 1,092 per genome. The RA and RD ranged from 7.6–8.4 and 53.0–59.5, respectively, in the analyzed ORFV genomes. However, previous reports in other DNA viruses such as human papillomaviruses (HPVs), the RA and RD ranged from 3.6–8.3 and 23.9–59.1^47^. In the case of Herpesviruses, RA and RD occurred to be 4.1–13.3 and 26.9–102.9^48^. On examining the SSR unit size classes, dinucleotide repeats were found to be most abundant (76.9%), followed by trinucleotide (17.7%) and mononucleotide repeats (4.9%) in all the genomes. Tetranucleotide and hexanucleotide repeats were least in number and represented 0.4% and 0.2% within the ORFV genome, respectively. There were no SSRs with pentanucleotide repeats observed in the ORFV genome. Approximately 90% and 10% of microsatellite motifs were distributed within coding and noncoding regions. Among the non-coding region, 4.8% are present in the UTR, while 5.4% in the intergenic regions, where functional protein and hypothetical protein occupied 68.8% and 21%, respectively. The genome-wide scan revealed the presence of 83–107 cSSRs, with an average of 93 occurrences per genome. In the case of compound microsatellites, the calculated RA and RD ranged from 0.6–0.8 and 12.1–17.0. However, in other DNA viruses such as HPVs, RA, and RD exhibited 0–1.2 and 0–27.3, whereas, in Herpesviruses, the RA and RD occurred 0.1–1.8 and 2.2–35.1^47,48^. Approximately 89.5% and 10.5% of microsatellite motifs were distributed within coding or non-coding regions, respectively. Among the non-coding region, 5.0% were represented in the UTR while 5.5% in the intergenic region, where functional protein and hypothetical protein occupied 60.7% and 28.8%, respectively (Figure S2).

**Figure 2:**
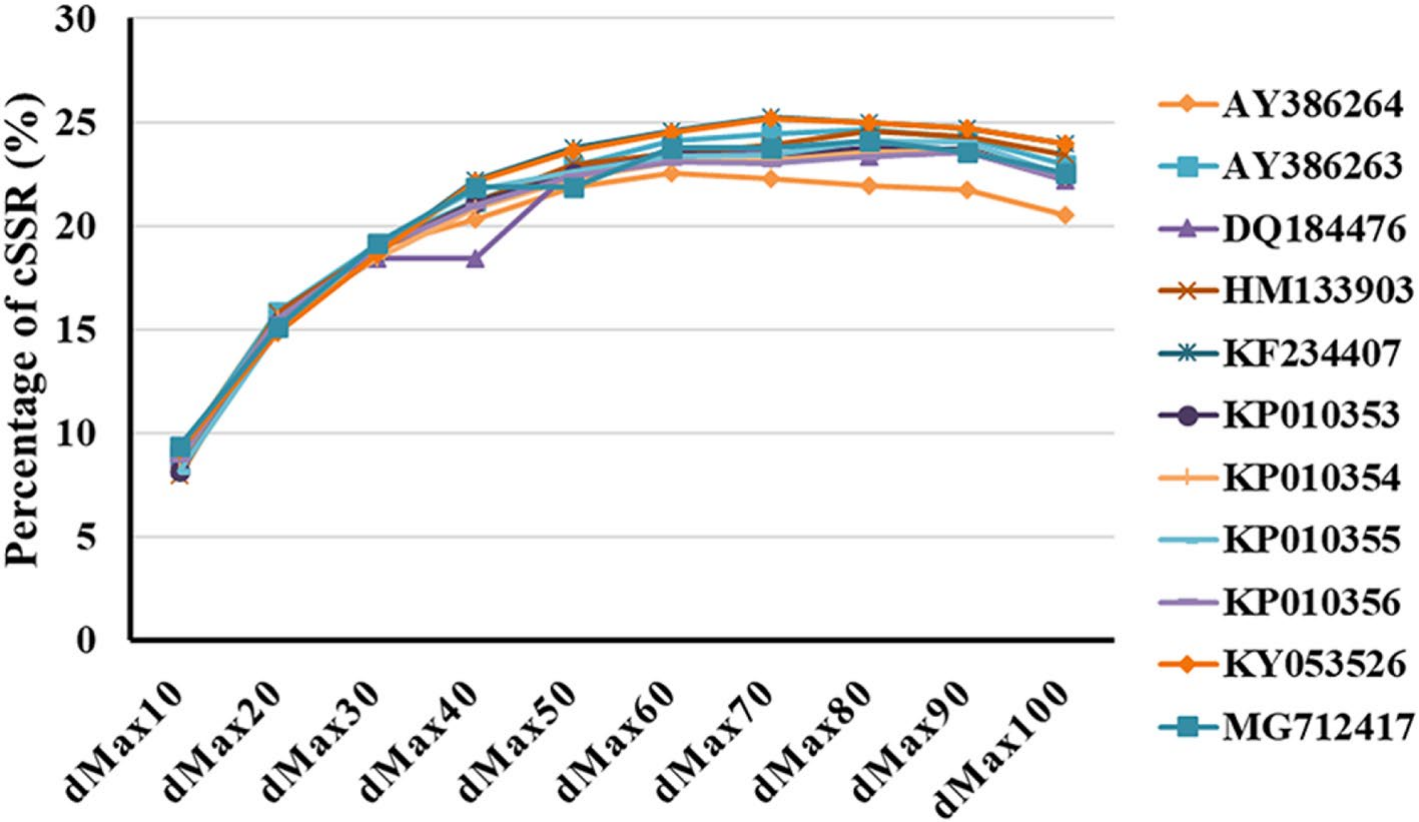
Frequency of cSSRs in relation to varying dMAX (10–100) across eleven ORFV complete genomes represented on the right side of the graph. A higher cSSR incidence was observed with increasing dMAX in the genomes.

The percentage of individual microsatellites being part of compound microsatellite (cSSR%) ranged from 7.9 to 9.0 (Table 1). Based on dMAX value, the maximum distance between any two adjacent microsatellites and if the distance separating two microsatellites is less than or equivalent to dMAX, than microsatellites are classified as cSSRs^49^. To determine the impact of dMAX, all the studied genome sequences were chosen to determine the variability of cSSRs with increasing dMAX. The value of dMAX was set between 10 and 100 by Microsatellite Identification Search Analysis (MISA)^50^. Our analysis revealed an overall increase in the number of cSSRs with higher dMAX value and attained a plateau (Fig. 2).

### Genomic parameters influencing SSR and cSSR distribution

We tested for the correlation between genome size and GC content with the incidence, RA, RD of SSRs and cSSRs. Except incidence (R^2^ = 0.6162, *p* > 0.05), all other parameters such as RA and RD of SSRs had no correlation (R^2^ = 0.002374, *p* > 0.05; R^2^ = 0.18, *p* < 0.05) with the genome size and GC content (R^2^ = 0.09377, *p* < 0.05, R^2^ = 0.00126, *p* > 0.05; R^2^ = 0.08129, *p* < 0.05). The regression analysis of cSSRs showed significant correlation with the incidence (R^2^ = 0.6483, *p* > 0.05) and RA (R^2^ = 0.4823, *p* > 0.05) while displayed non-significant correlation with RD (R^2^ = 0.3759, *p* < 0.05). On the contrary, the GC content was weakly correlated with the number (R^2^ = 0.02903, *p* > 0.05), RD (R^2^ = 0.004839, *p* < 0.05) and RA (R^2^ = 0.03917, *p* < 0.05) of cSSRs.

### The frequency of classified repeat types

**Figure 3:**
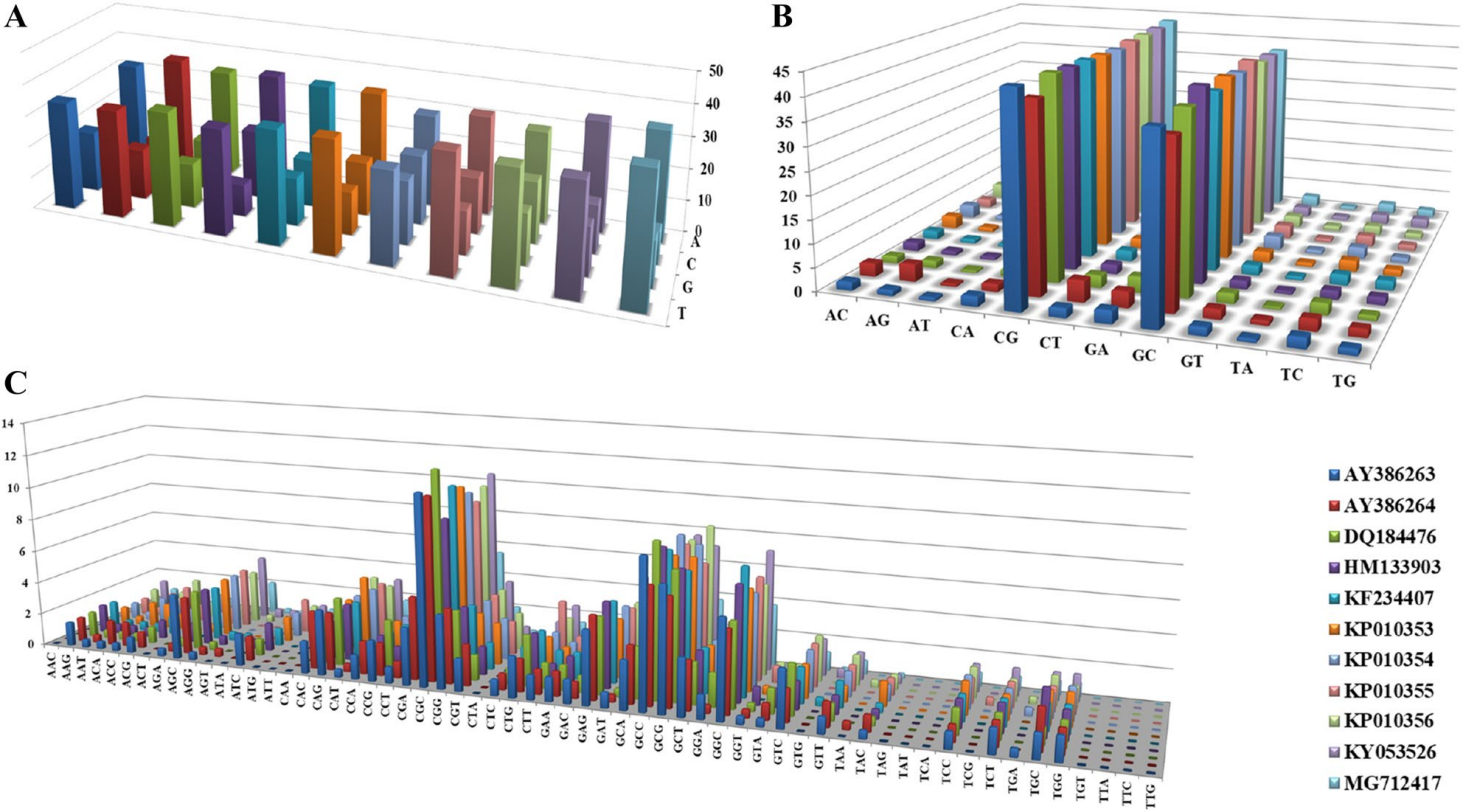
Types of SSRs distribution. (**A**) Distribution of different motifs of mononucleotide SSRs within ORFV genomes, (**B**) distribution of different motifs of dinucleotide SSRs within ORFV genomes, and (**C**) distribution of different motifs of trinucleotide SSRs within ORFV genomes.

The overall frequency of mononucleotide repeats A/T (64.1%), dinucleotide repeat motif CG/GC (81.6%) were the most prevalent than poly G/C (35.9%), GA/TC (5.0%), AC/GT (4.5%), AG/CT (3.9%), CA/TG (3.6%) and AT/TA (1.4%), respectively. Analysis of the classified tri-repeat types revealed that the ORFV genome had 30 types of trinucleotide from which CGC/GCG, GCC/GGC, CAG/CTG, AGC/GCT, CCG/CGG were abundantly present exhibiting 18.2%, 14.5%, 6.3%, 6.2%, and 6.3%, respectively. The most common tetra and hexanucleotide repeats were CGAG/CTCG (34.9%), ACTC/GAGT (18.6%), GTGA/TCAC (9.3%) and AGTTAC/GTAACT (15.0%), ACACTC/GAGTGT (15.0%), respectively. However, the accession specific analysis illustrated that the frequency of mono, di, tri repeats varied from each other (Fig. 3a–c).

### Motif complexity of compound microsatellites

Compound microsatellites (cSSRs) are composed of two or more adjacent individual microsatellites. Generally, cSSR having the pattern like, m1-xn-m2, m1-xn-m2-xn-m3 are considered as ‘2-microsatellite’ and ‘3-microsatellite’, respectively^49^. Majority of cSSRs were composed of two motifs, followed by tri, tetra, and penta-motifs (Supplementary file 1). Interestingly, two long stretches of cSSR were composed of identical motifs repeated 12 times, which was exclusively found in the genome of AY386264. The CTG–CAG compound microsatellite composed of self-complementary motifs has been proposed to be created by recombination^51^. However, our study showed no such compound microsatellites which contained self-complementary motifs, suggesting that these compound microsatellites were not likely to be derived from recombination. Motifs exhibiting the form [m1]n-xn-[m2]n can be termed as SSR-couples and are represented the maximum time in the genome. In this study, SSR couples, such as (CG)-x-(GC), (GC)-x-(GC), (GC)-x-(CGC), (GT)-x-(GC), (GC)-x-(CG), (CT)-x-(C) were presented in all analyzed genome. A number of self-complementary motifs such as (CG)3-x1-(GC)3, (CG)4-x1-(GC)3, (CG)3-x7-(GC)3, (CG)3-x0-(GC)3, (GC)3-x8-(CG)3, (CG)3-x7-(CG)3, (GC)3-x0-(CG)3, (CG)3-x4-(GC)3 have been observed in ORFV, which played a pivotal role in secondary structure formation. Motif duplication is one of the phenomena in which a similar motif is located on both ends of the spacer sequence, for example (CA)n-(X)y-(CA)z. About 22.1% of the total cSSR were made up of duplicated sequences having the motif pattern (GC)-x-(GC), (CG)-x-(CG), (GA)-x-(GA), (CA)-x-(CA), (CT)-x-(CT), (TC)-x-(TC), (CA)-x-(CA)-x-(CA), (A)-x-(A), (AG)-x-(AG)-x-(AG)-x-(AG)-x-(AG)-x-(AG)3-x-(AG)-x1-(AG)-x-(AG)-x-(AG)-x-(AG)-x-(AG)-x-(AG), (AG)-x-(AG), (C)-x-(C), (CA)-x-(CA), and (CT)-x-(CT)-x-(CT)-x-(CT)-x-(CT)-x-(CT)-x-(CT)-x-(CT)-x-(CT)-x-(CT)-x-(CT)-x-(CT)-x-(CT) (Supplementary file 1).

### Identification of polymorphic microsatellites through in silico approach

**Figure 4:**
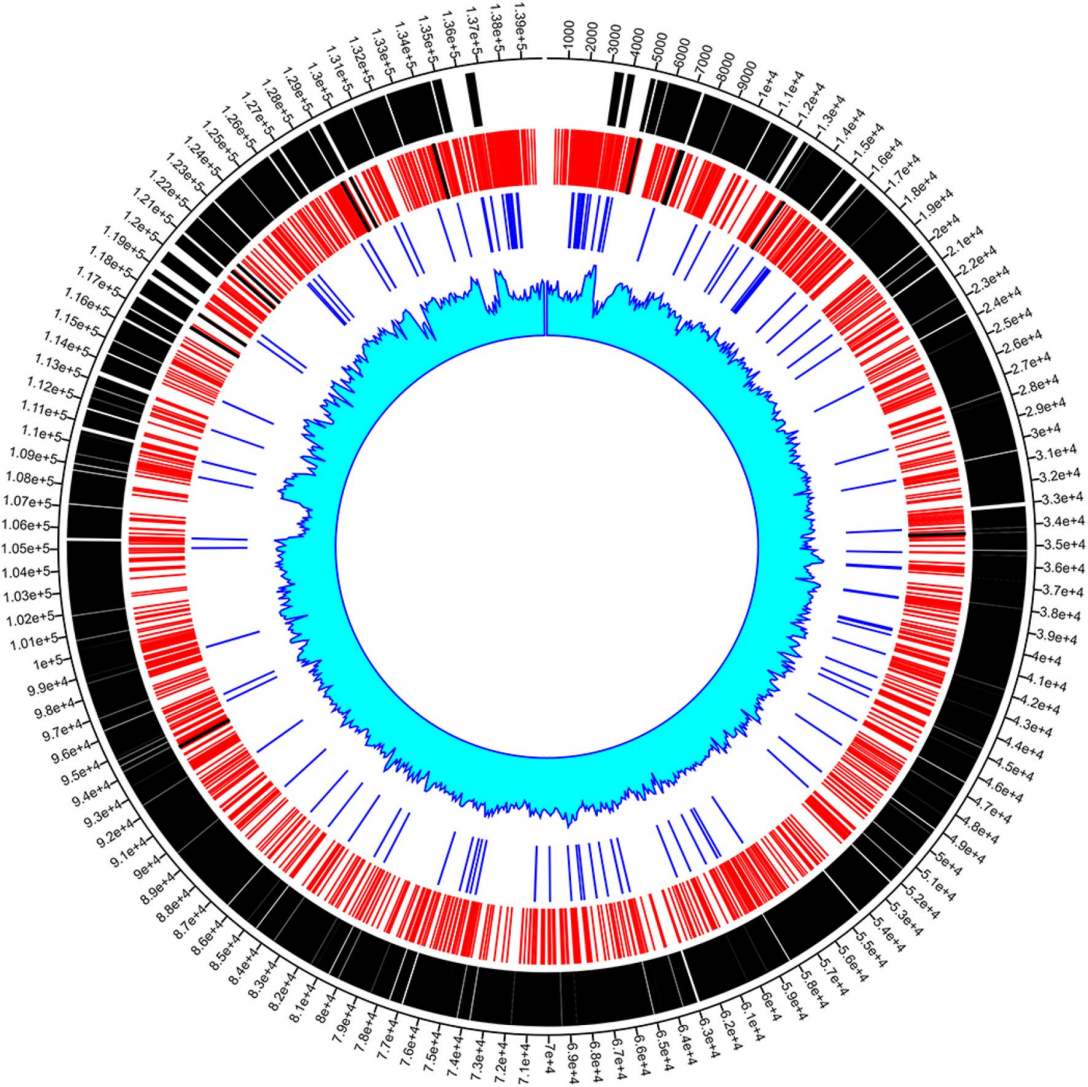
Circos plot showing the Genome size, CDS, Distribution of SSRs, selected SSR markers, cSSRs and GC content in ORFV (OV-SA00) genome. From outer track to inner track: Genome size, CDS, SSRs, selected SSR markers (Black lines within the SSR), cSSRs and GC content.

For a polymorphic microsatellite, the length of the repeat block should be non-identical with that of the other sequences in the database, and this length difference must be a multiple of the repeat unit^19,30,52^. For the identification of a polymorphic microsatellite, eleven strains of ORFV were used, where (AY386264) acted as the reference. A total thirteen number of polymorphic microsatellites were observed; among these, two were observed within the hypothetical protein, three in the intergenic regions, and rest eight in the protein-coding/genic regions. The polymorphic genic region containing the microsatellites encodes several important proteins such as Ankyrin repeat protein (ANK protein), DNA-binding phosphoprotein, virion core protein, Granulocyte–macrophage colony-stimulating factor (GM-CSF), Interleukin 10 protein (IL-10), Putative serine/threonine-protein kinase protein (Table 2, Figure S3). The Circos map provides a clear vision regarding the SSRs and cSSRs distribution and other related details in ORFV (OV-SA00) genome (Fig. 4).

### Development and characterization of SSR markers

**Figure 5:**
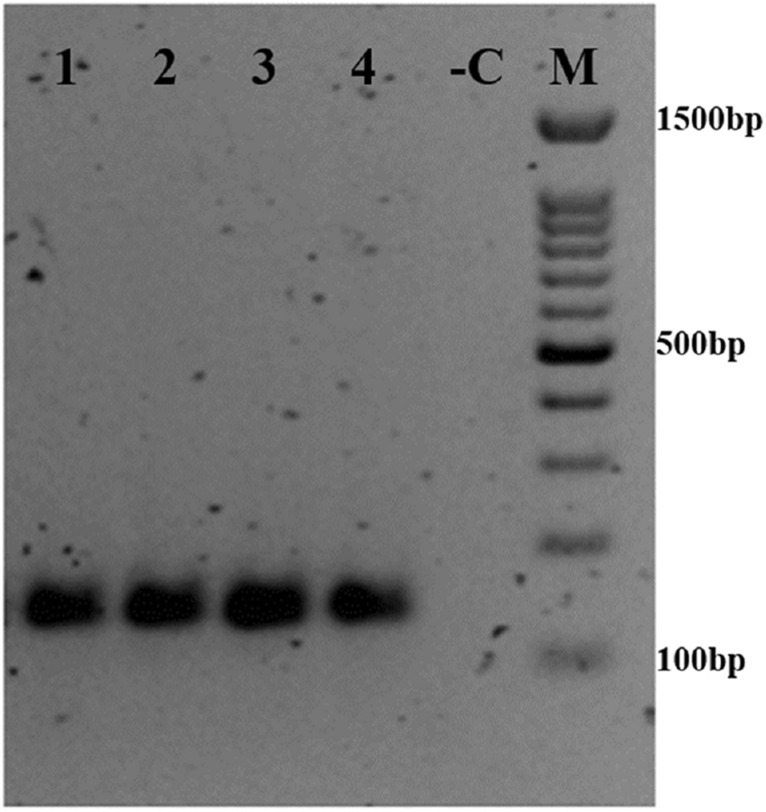
Clinical samples evaluation by universal OFRV primers. Electrophoresis gel showing the PCR amplicon of four suspected ORFV clinical samples collected from Black Bengal goats. M: 100 bp DNA ladder; -C: Negative control (PCR using nuclease-free water as DNA template); 1–4: Clinical samples.

All clinical samples collected during the outbreak were found to be positive for ORFV tested by producing the desired PCR amplicon size of 140 bp (Fig. 5).

**Figure 6:**
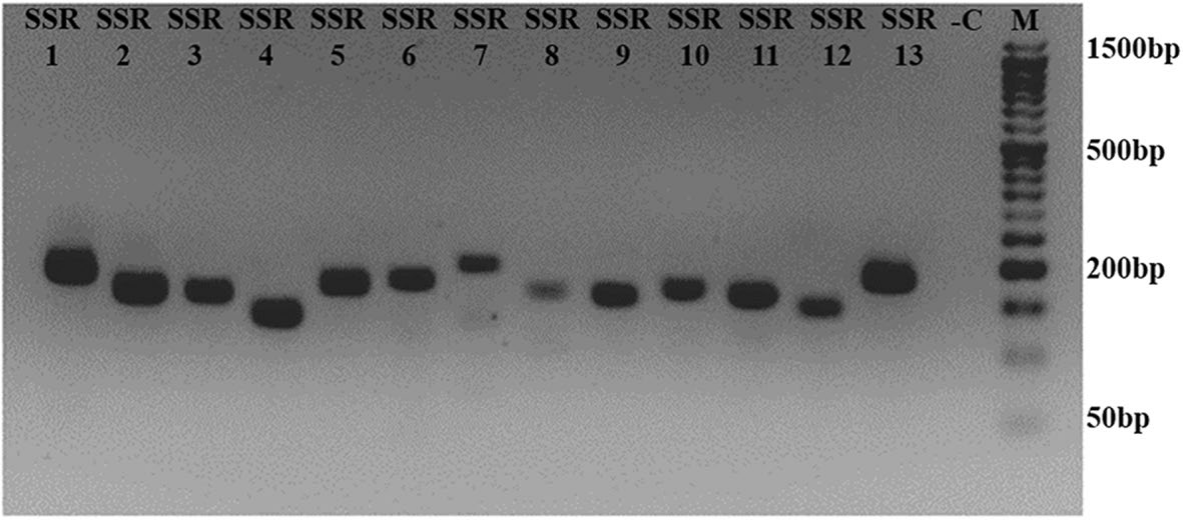
Clinical sample validation using SSR markers. Electrophoresis gel showing the PCR amplicon of the developed SSR markers in ORFV. SSR markers from SSR1 to SSR13; M:50 bp DNA ladder; -C: Negative control (PCR using nuclease-free water as DNA template).

We chose all thirteen polymorphic markers to validate in-vitro. Hence, PCR was set with each primer sets to amplify the DNA isolated from a positive clinical sample. The SSR name, primer sequences, expected size, targeted motif, functional region, protein motif position, gene, ORF number, and annealing temperature, were summarized in Table 2. All the SSR markers produced reliable and reproducible PCR products with the expected molecular size (Fig. 6).

The amplified SSRs were further characterized by sequencing, mapping with the GenBank database through BLASTn and BLASTx. The results of BLASTn alignment revealed a 100% of query coverage and a high identity percentage (91–100%) between the respective sequencing product and their equivalent genes from the published OV-SA00 isolate genome sequence. The results of BLASTx alignment revealed various degrees of query coverage (38–96%) and a high identity percentage (91–100%) with their equivalent amino acid sequences (Table 3).

**Table 3:** Alignment of the 13 sequenced microsatellite markers (partial) against the complete genome present in the NCBI database.

SSR	BLASTn			BLASTx		
	Query cover (%)	E value	Identity (%)	Query cover	E value	Identity
ORFV-SSR-1	100	6.00E−81	96	52%	0.41	91%
ORFV-SSR-2	100	5.00E−76	100	81%	2.00E−19	100%
ORFV-SSR-3	100	2.00E−50	91	Intergenic	Intergenic	Intergenic
ORFV-SSR-4	100	5.00E−60	100	96%	1.00E−18	100%
ORFV-SSR-5	100	6.00E−80	99	55%	3.00E−12	100%
ORFV-SSR-6	100	1.00E−67	95	41%	6.00E−05	100%
ORFV-SSR-7	100	2.00E−85	97	Intergenic	Intergenic	Intergenic
ORFV-SSR-8	100	5.00E−65	92	65%	2.00E−18	100%
ORFV-SSR-9	100	4.00E−67	97	67%	5.00E−17	100%
ORFV-SSR-10	100	6.00E−75	99	71%	2.00E−07	100%
ORFV-SSR-11	100	2.00E−74	99	98%	9.00E−30	100%
ORFV-SSR-12	100	2.00E−78	92	Intergenic	Intergenic	Intergenic
ORFV-SSR-13	100	3.00E−23	100	38%	1.00E−15	100%

**Figure 7:**
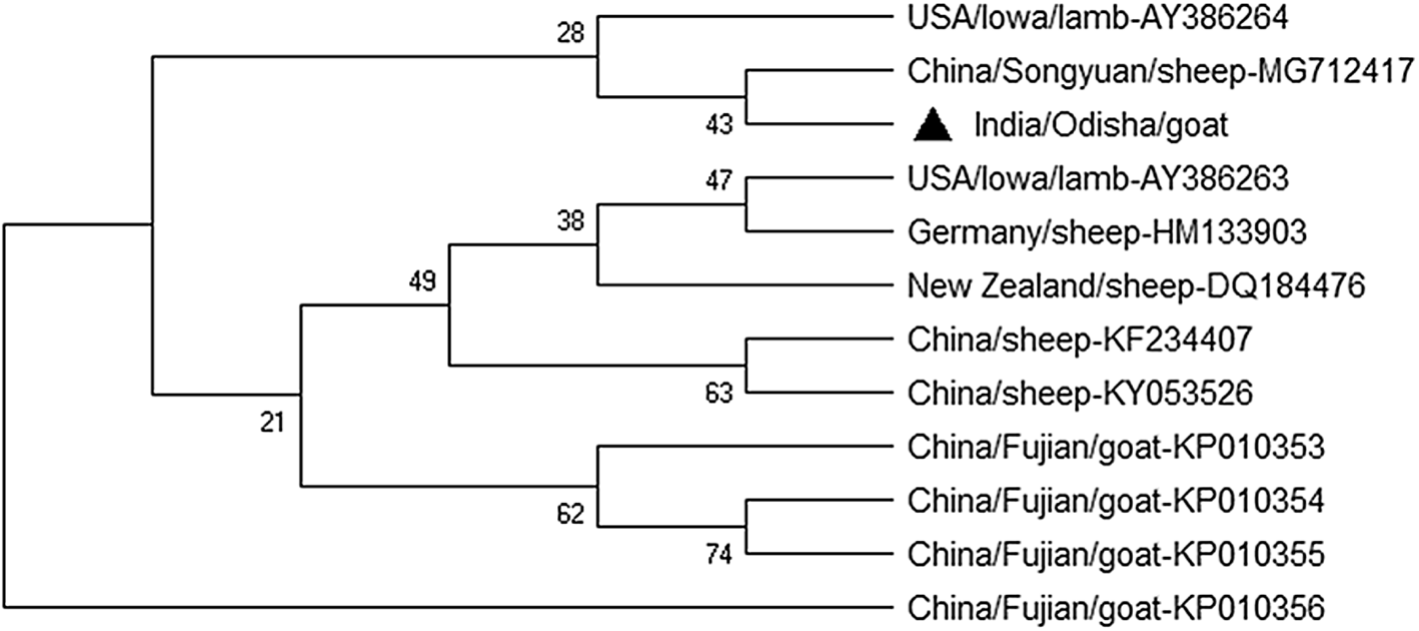
The concatenated phylogenetic tree was constructed using the bootstrap consensus tree building method of neighbor-joining with bootstrap value 500 using MEGA 5. Black triangle represents the ORFV isolates of present investigation showing its relationship with eleven global strains.

The concatenated phylogenetic tree showed the ORFV of our study closely related to Chinese isolate (MG712417) (Fig. 7). We observed the presence of 2–3 alleles within ORFV genomes.

## Discussion

Microsatellites, otherwise known as short tandem repeats (STRs), or a variable number of tandem repeats (VNTRs) are being used to discriminate various viruses, such as human cytomegalovirus (hCMV)^22,23^, white spot syndrome virus (WSSV)^53–58^, Herpes Simplex virus type 1^30,59^, Herpes Simplex virus type 2^60^, Herpesvirus 3^61^, Herpesvirus 6^62^, Adenovirus^63^, Ostreid herpesvirus 1^64,65^, Marek’s disease virus 1^66^, and Spodoptera littoralis multiple nucleopolyhedrovirus (SpliMNPV)^67^ due to its polymorphic in nature. To get the insight into the microsatellite in ORFV, we have employed a comparative genomics approach for development and characterization through in-silico and in-vitro analysis and validated our findings using samples collected from the recent Orf outbreak for the first time.

The specific parameters, such as its incidence, RA and RD of SSR and cSSR in ORFV genomes, show abundance variation as compared to their genome size and GC content due to the heterogeneity of ORFVs. Until now, limited full-length ORF genomes exist in the database. Based on our analysis, we observed little variation in RA and RD in ORFV. However, in other viruses such as HPVs^47^ and Herpesviruses^48^, higher variation in RA and RD were reported. The large variation with the parameters was not observed in ORFV, probably due to the lack of enough size difference in the genome. However, a limited number of complete genome sequences are available for this virus, in comparison to HPV and herpesviruses, which act as a constraint to get the optimal range. Correlation analysis confirmed that incidence of both SSRs and cSSRs, RA of cSSRs were dependent on genome size, but independent of GC content, which was similar to that of HPV^47^, but opposite to HIV^68^, potexvirus, carlavirus, and tobamovirus^69–71^. The distribution of microsatellite in the viral genome is pathogen-specific rather than host-specific. The increase of cSSR is predominant when dMAX approaches 10–90 bp and further decreases with the increase of dmax (Fig. 2). This may be due to the occurrence of SSR in the overlapping regions of increasing dMAX. The ORFV genomes have more SSR within coding regions than non-coding regions in comparison with other DNA virus, such as herpes simplex virus. This might be due to higher relaxed selection pressure on coding regions in comparison to the non-coding region in the respective virus.

The cSSRs percentages of ORFV ranges from 7.9 to 9.0%, which is lower in comparison to HIV-1, 0–24.2%^68^, Geminivirus, 0–27.2%^72^, Herpesvirus, 8.1–33.3%^48^. Generally, the number of compound microsatellites decreases with an increase in complexity^73^. Moreover, the lack of sufficient genomic resources from diverse geographical locations may contribute to a stagnant range of cSSRs%. In ORFV, 22.1% of cSSRs were composed of similar motifs, probably contributed by genome duplication. Some study suggests that genome duplication may be helpful for the repeat tendency mechanism^74^, which promotes the expansion of genome size such as yeast^75,76^.

In ORFV genomes, the poly A/T repeats were significantly more prevalent than poly G/C repeats, similar to eukaryotic and prokaryotic genomes^1,2^. The presence of mononucleotide repeats in Mengovirus and Encephalomyocarditis virus affect virus growth in murine cell culture^77^. In the case of ORFV, its significance needs further validation. In this study, we also observed the microsatellites having polymorphism in poly A/T (ORF117), poly C/G (ORF121), within the important immune-regulatory genes, such as in GM-CSF and ANK protein, respectively (Supplementary file 2). GM-CSF secreted by a variety of cell types triggers neutrophil, monocyte, and eosinophil myelopoiesis and stimulate early events in immune responses, controlling the differentiation and function of antigen-presenting dendritic cells. IL-2 is a T-cell-derived lymphokine that stimulates T-cell and NK cell activation and proliferation and activated-B-cell proliferation^78,79^. ANK protein leads to the down-regulation of hypoxia-induced factor (HIF) activity and regulates energy metabolism, angiogenesis, the apoptotic cascade, the *NF-kB* signaling pathway, and cell cycle regulation^80^. The functional effects of this polymorphism in these regions require further investigations.

Dinucleotide CG/GC is more prevalent in most of the ORFV genomes, similar to that of DNA viruses such as HPVs^47^, Caulimoviruses, Geminiviruses^52,81^. CG/GC repeat could form Z‐conformation or other alternative secondary DNA to facilitate the recombination activity^82^. In our study, the polymorphism within dinucleotide (AC/CA)3 and (CG/CG)3 observed within the hypothetical protein. Dinucleotide repeats have the highest slippage rate as compared to any other type of repeats^81^. Among 257 viral genomes examined in a published study, the highest number of dinucleotide SSRs were found when compared to the other types^83^. Dinucleotide repeats are also speculated to be recombination hot spots^84,85^. In this study, the presence of higher di-nucleotide repeats over tri-nucleotide repeats suggests a possible role of hosts in the evolution of di-nucleotide repeats within poxvirus genomes. Inconsistency frequency of SSRs in different accession of the same virus may be attributed to instability because of a higher slippage rate^86^.

Trinucleotide motif ATA/TAA/AAT or ATT/TTA/TAT were most prevalent in most genomes of poxvirus whereas in other DNA virus GAG/AGA was most prevalent in HPVs and AAG/GAA in caulimoviruses. The higher density of trinucleotide repeats was observed compared to any other repeat type within coding regions of eukaryotic and prokaryotic genomes^32^. Interestingly, dynamic mutations within trinucleotide repeats responsible for the development of some diseases in humans^87^, as well as viral enzymes that interfere pathogenicity of Influenza virus^88^. Our study revealed the presence of trinucleotide CGC/GCG and GCC/GGC repeats to be most prevalent than others. The trinucleotide polymorphism was observed in some immunoregulatory genes such as ANK protein (GGC/GCC)_3_ (ORF008), IL-10 protein (AGT/ACT)_3_ (ORF127) and structural genes virion core protein (GAG/CTC)_3_, Putative serine/threonine-protein kinase (CGC/GCG)_3_, which needs further functional evaluation.

Three polymorphic SSRs such as (A/T)_7_, (T/A)_6_, (AGTTAC/ GTAACT)_3_ were observed within non-coding regions. The microsatellite present within the non-coding reasons was evolutionarily neutral and can be utilized as an excellent molecular marker^30^. Finally, we have characterized, those polymorphic markers present at non-genic as well as coding (genic) regions. These genic microsatellites, however, may provide adaptive variation important to viral evolution and genetic variability, perhaps similar to the functionally important mononucleotide runs found in VSV^34^ and RSV^36^ and virulence of avian influenza virus encephalo-myocarditis virus^89,90^. It is noteworthy to mention that, recently, the microsatellite present in HSV-1 glycoprotein coding region US4 was useful for strain differentiation^30^. The concatenated tree, which was constructed utilizing sequence information of characterized markers, confirmed that the ORFV of the present study closely related to Chinese isolate (MG712417). Our previous report, as well as several other studies, observed a similar pattern of relationship^18,91^. We speculate that trans-boundary and cross-species transfer of ORFV isolates could have resulted in this, as India is geographically adjacent to China. It is interesting to observe the presence of a number of the alleles (2–3) within ORFV genomes indicates the existence of polymorphism within microsatellites, which could act as a useful tool to estimate the diversity^61^. Using a single repeated mononucleotide was able to follow the dynamics of transmission of a human adenovirus during an epidemic^63^. Therefore, microsatellites constitute a potentially powerful tool for epidemiological studies of the transmission routes and evolution of ORFV and other related poxviruses. This study provides an important new type of molecular markers useful to investigate questions not only related to epidemiology but also for deciphering the diversity of the virus. However, the characterized microsatellites of the present study are not biased to the particular strain, which indicates the presence of recombinant strains circulating within the Indian subcontinent. This information is not concrete, which requires validation by several whole-genome sequence analysis of ORFV isolates from Indian origin. So far, our understanding of the functional and evolutionary role of microsatellites in ORFV biology is limited, which needs further in-depth evaluation and possible implementation.

In conclusion, the study of microsatellites in ORFV genome is a key step towards better understanding the nature, function, and evolutionary biology of the species. Our preliminary results can be considered as a useful tool for ORFV strain demarcation, diversity estimation, and evolutionary analysis. Our next plan is to characterize several ORFV strain complete genome from Indian origin through next-generation sequencing to get a better insight into genome organization, development of a suitable multiplex panel, which can be utilized as an effective tool for virus identification, genotyping and evolutionary analysis of the respective virus.

## Supplementary information

Supplementary Information 1.

Supplementary Information 2.
