# Supplementary material for: Comparative analysis, distribution, and characterization of microsatellites in Orf virus genome
Source: Sci Rep. 2020 Aug 17;10:13852. doi: 10.1038/s41598-020-70634-6 (PMC7431841; doi:10.1038/s41598-020-70634-6)
Supplement: Supplementary file 1 — Supplementary Information 1. [file 41598_2020_70634_MOESM1_ESM.docx]

**Supplementary File 2**

**Comparative analyses, distribution, and characterization of microsatellites in Orf virus genome**

B. P. Sahu^a^, Prativa Majee^a^, R.R Singh^a^, A. Sahoo^b^, D. Nayak^a^

^a^Indian Institute of Technology Indore, MP 453 552, India

^b^College of veterinary science and animal husbandry, Bhubaneswar, 751003, India

* Corresponding author: Email: nayakdn[at]iiti.ac.in

**Supplementary Figure 1:** Distribution of SSR. Pie chart illustrating the percentage of differential distribution of SSR within the coding (UTR and intergenic region) and non-coding (functional and hypothetical proteins) regions of ORFV genome.


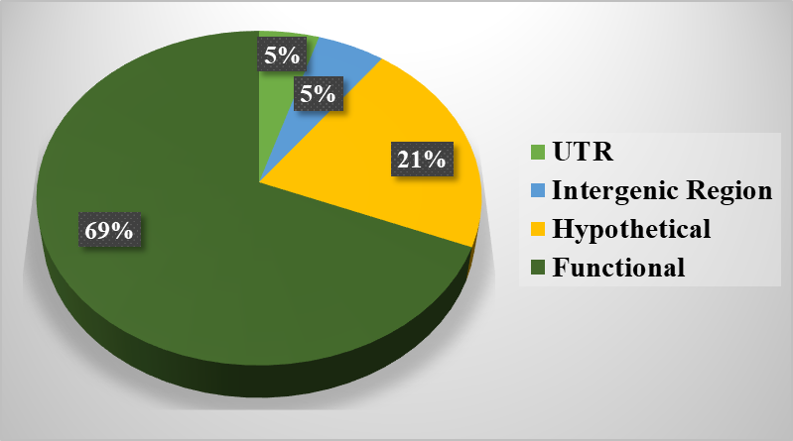


**Supplementary Figure 2:** Distribution of cSSR. Pie chart illustrating the percentage of differential distribution of cSSR within coding (UTR and intergenic region) and non-coding (functional and hypothetical proteins) regions of ORFV genomes.


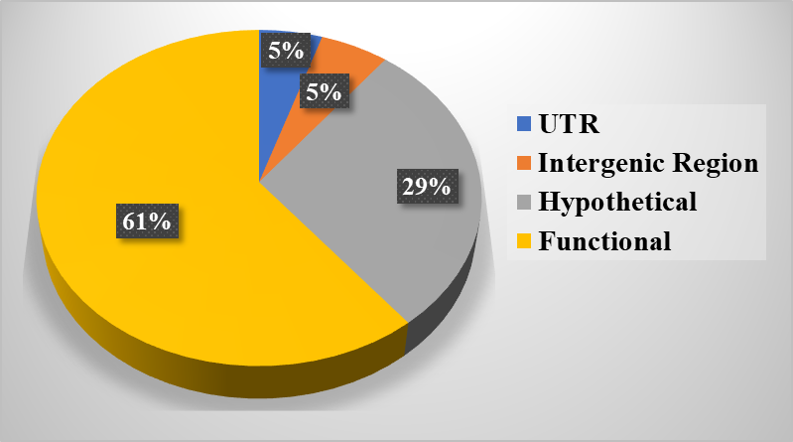


**Supplementary Figure S3:** Polymorphism observed within SSR markers of ORFV genomes.


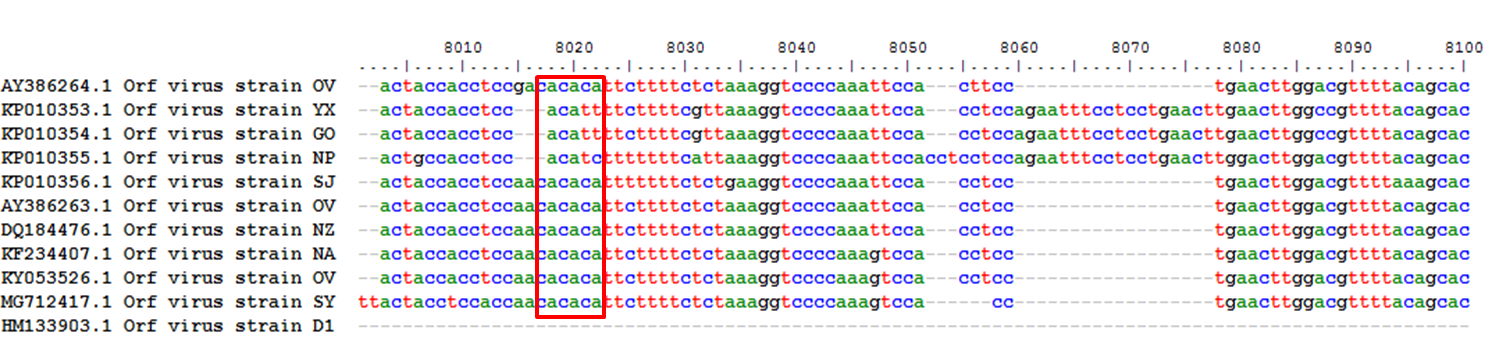
**Figure S3a:** SSR1


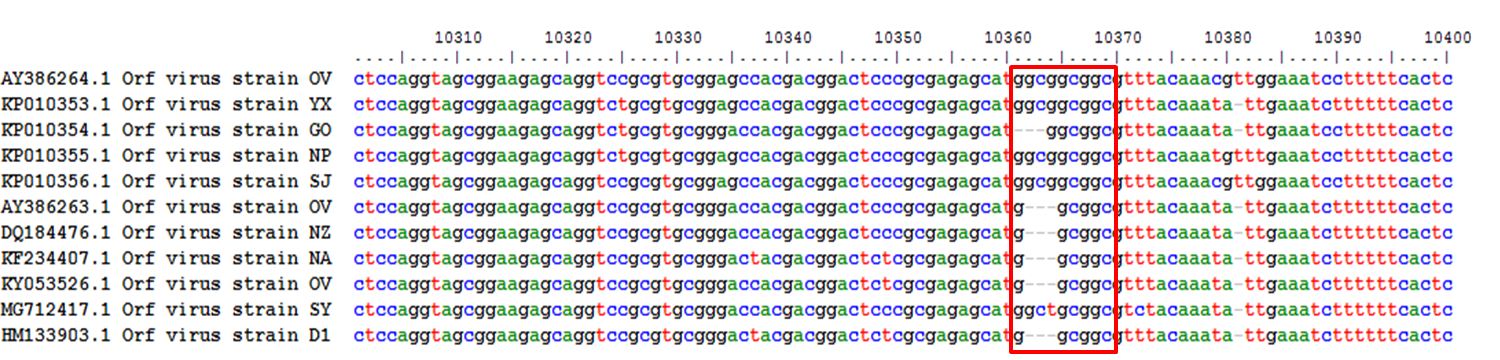
 **Figure S3b:** SSR2


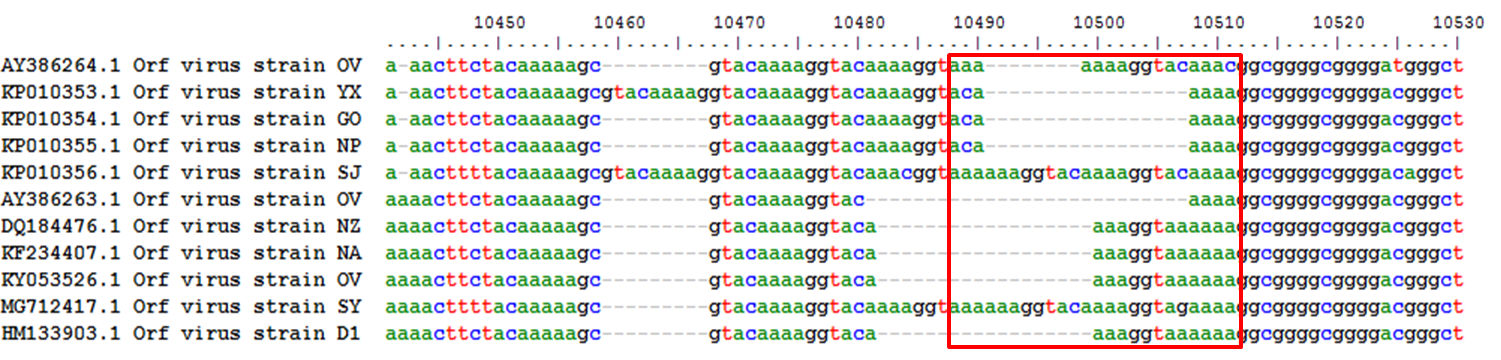
 **Figure S3c:** SSR3


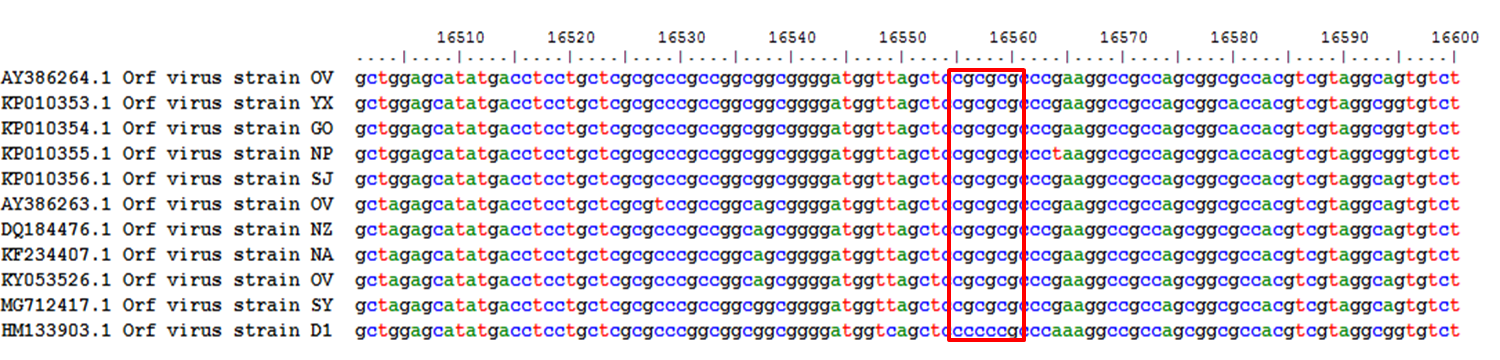
 **Figure S3d:** SSR4


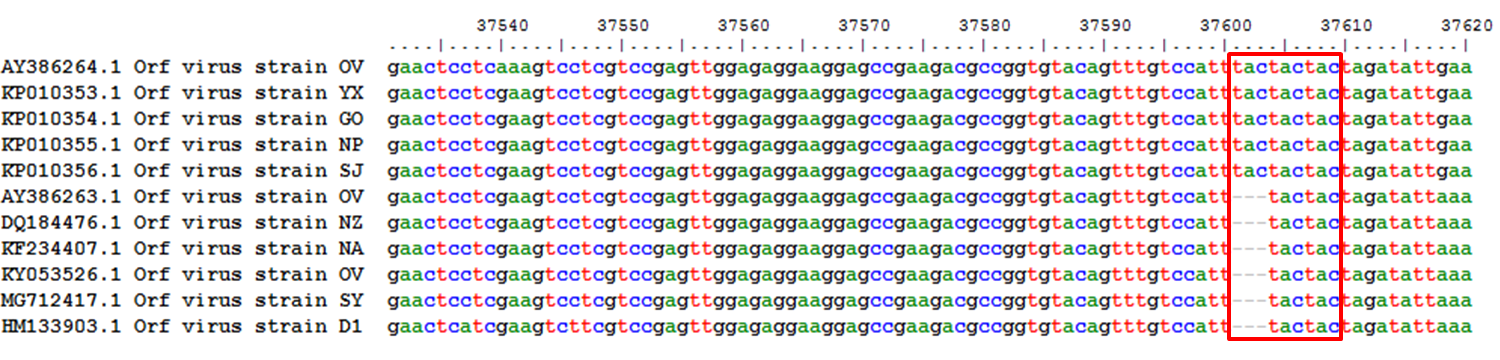
 **Figure S3e:** SSR5
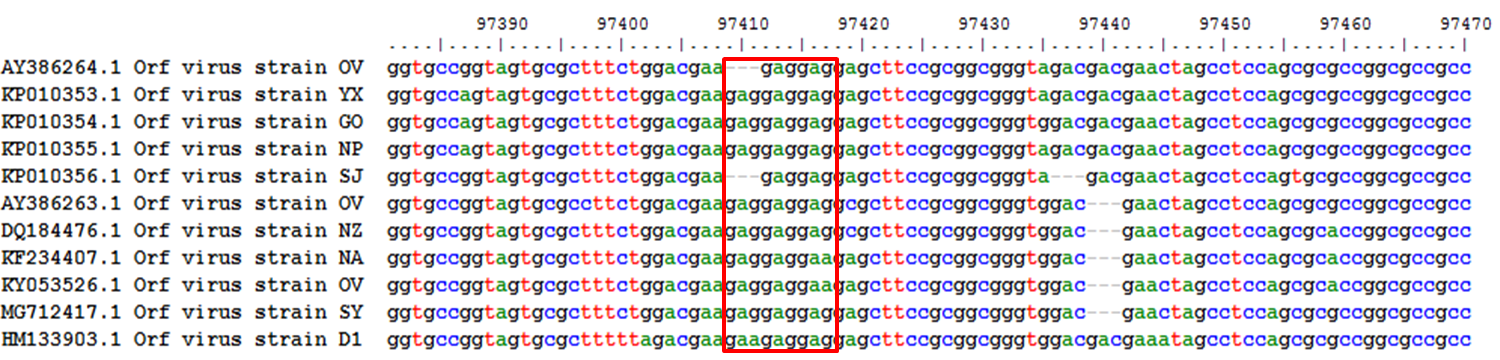
 **Figure S3f:** SSR6


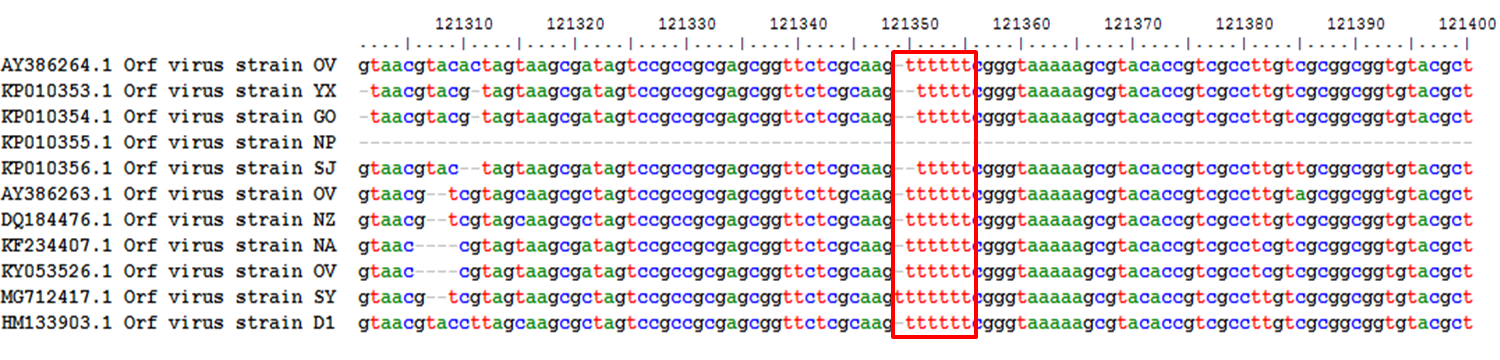
 **Figure S3g:** SSR7


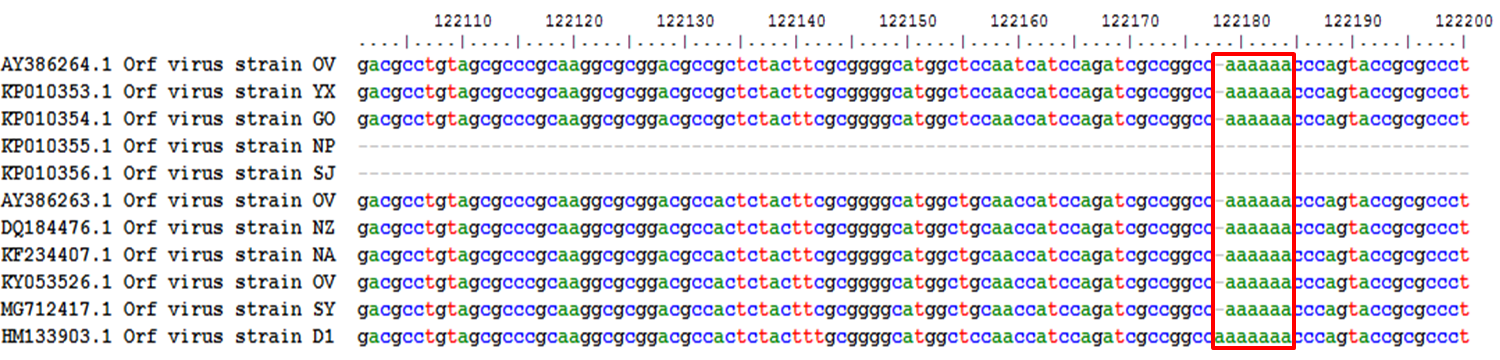
 **Figure S3h:** SSR8


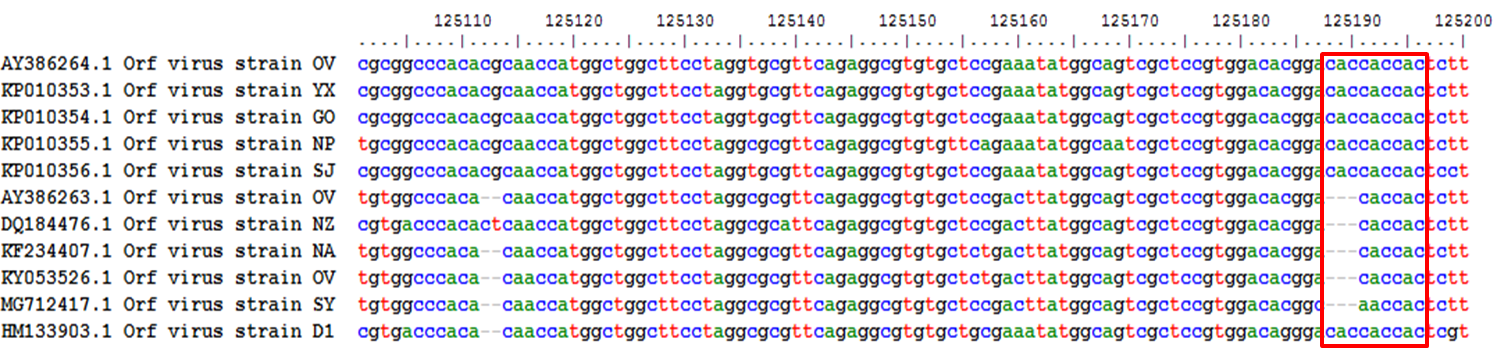
 **Figure S3i:** SSR9


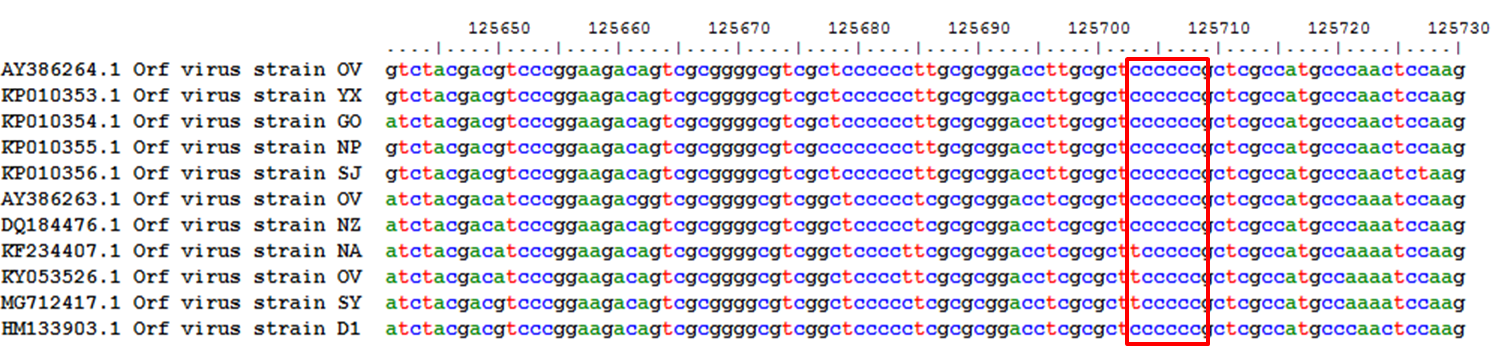
 **Figure S3j:** SSR10


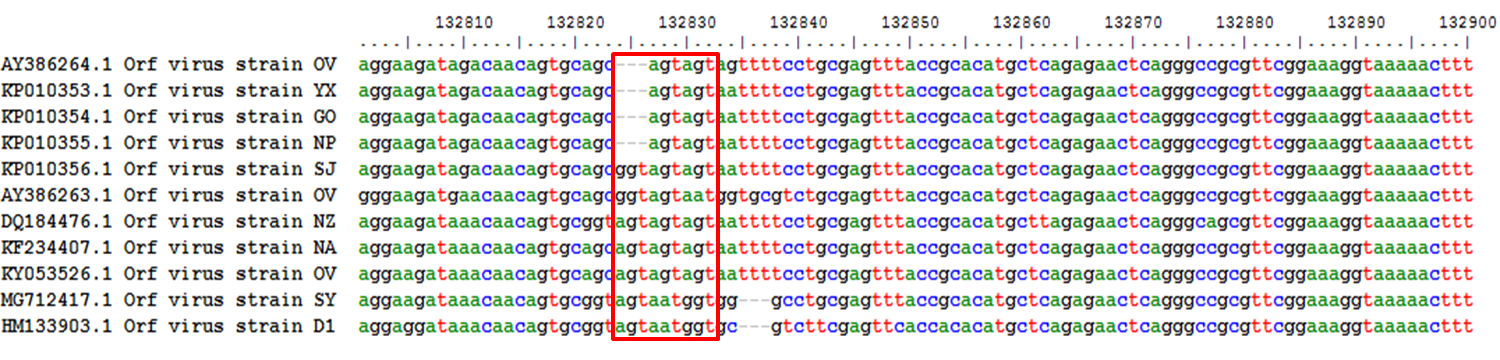
 **Figure S3k:** SSR11


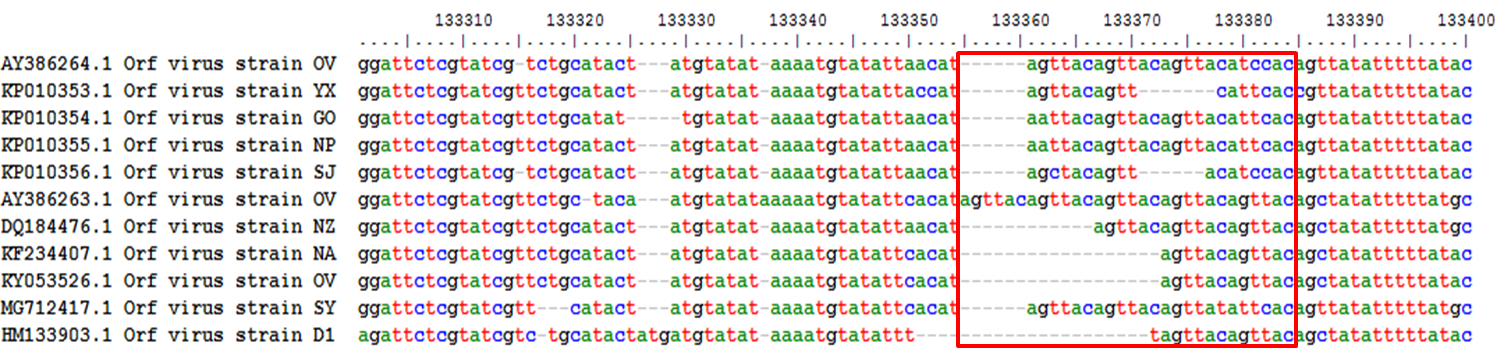
 **Figure S3l:** SSR12


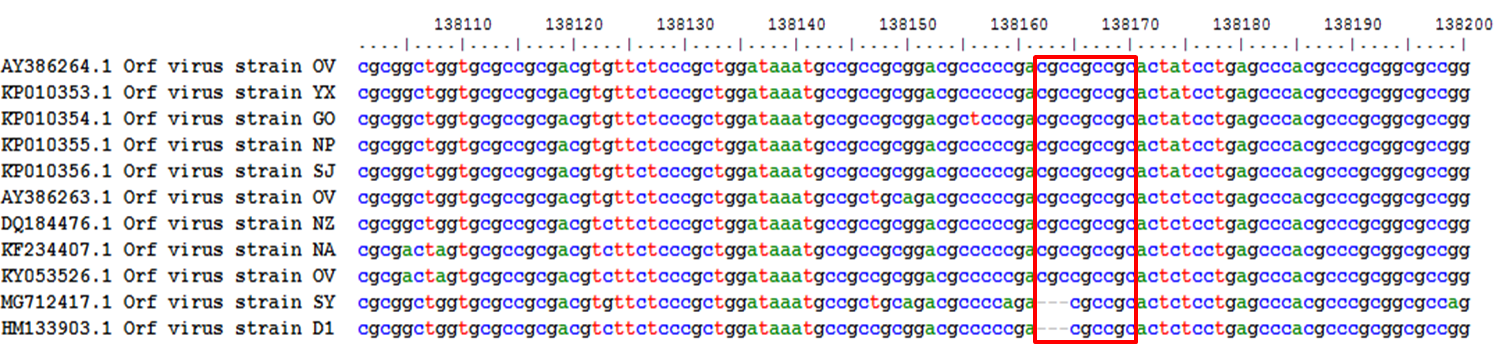
 **Figure S3m:** SSR13
